# Symptomatology of Uræmia

**Published:** 1881-04

**Authors:** 


DR. E. C. SEGUIN, in *Archives of Medicine*, Vol. 4, No. 1, August, 1880, contributes to the symptomatology of Uræmia, by an interesting summary of two cases in which occipital headache was so localized and persistent as to give rise to a strong suspicion of organic disease of the cerebellum, and in one (No. 2) a positive conclusion was only reached by means of a post mortem examination. Both cases appear to be cases of contracted kidneys.

Both patients were adults aged thirty-six and forty-seven. Both had suffered more or less from chronic headache of the migraine type. At a given period the headache became transformed into a localized occipital *pain* very different from the former attacks. This peculiar headache was distinctly paroxysmal, but not at all periodical. Both cases were accompanied by nausea. In neither was their suspicion of syphilis. Case No. 1

made relatively clearer by a previous history of convulsions and albuminuria four years ago; other symptoms of renal disease not marked; no ophthalmoscopic lesion; no dyspepsia; heart normal. Urine contains five per cent. albumen; sp. gr. 1018. Hyaline and granular casts.

Case 2 died in comatose state. No convulsions or paralytic symptoms having shown themselves though the pain had extended to sixth cervical vertebra. Urine examined four days before death gave ten per cent. albumen with numerous hyaline and granular casts. Autopsy showed extensive granular disease; cerebellum and brain in normal condition.

While in some respects the story of these cases is imperfect, and Dr. S. particularly regrets lack of observation upon the quantity of urine passed and the state of arterial tension, yet he is inclined to believe that their publication may serve to render more accurate the diagnosis of occipital headache and illustrate the utility of critically examining the urine in cases of any degree of obscurity, especially as occipital headache is scarcely mentioned as a symptom of uræmia.

